# Formulation and Evaluation of Mouth Dissolving Tablets of Cinnarizine

**DOI:** 10.4103/0250-474X.73930

**Published:** 2010

**Authors:** B. P. Patel, J. K. Patel, G. C. Rajput, R. S. Thakor

**Affiliations:** Nootan Pharmacy College, S. P. Sahakar Vidyadham, Visnagar-384 315, India

**Keywords:** Cinnarizine, *In vitro* disintegration time, mouth dissolving tablets, sublimation, wetting time

## Abstract

The purpose of this research was to develop mouth dissolve tablets of cinnarizine by effervescent, superdisintegrant addition and sublimation methods. All the three formulations were evaluated for disintegration time, hardness and friability, among these superdisintegrant addition method showed lowest disintegration time; hence it was selected for further studies. Further nine batches (B1-B9) were prepared by using crospovidone, croscarmellose sodium and L-HPC in different concentrations such as 5, 7.5 and 10%. All the formulations were evaluated for weight variation, hardness, friability, drug content, *in vitro* disintegration time, wetting time, *in vitro* dissolution. Formulation with 10% L-HPC showed the less disintegration time (25.3 s) and less wetting time (29.1 s). *In vitro* dissolution studies showed total drug release at the end of 6 min.

Mouth dissolving tablet disintegrate or dissolve in saliva and are swallowed without the need for water. They offer an advantage over swallowing tablets and capsules. Difficulty to swallow is particularly experienced by pediatric and geriatric patients. Technique that are frequently employed in the preparation of mouth dissolving tablets include, freeze drying, sublimation, spray drying, moulding, mass extrusion and direct compression[1].

Cinnarizine is a H1-receptor antagonist that is widely used in the treatment of motion sickness, vomiting and vertigo. It is water insoluble and tasteless[2]. Hence it was select as a model drug for the preparation of mouth dissolving tablets. In the present work effervescent, superdisintegrant addition and sublimation technique were tried for formulation of tablets. Superdisintegrant addition method was found as best and further study carried out using three superdisintegrants in different ratios.

Cinnarizine was received as a gift sample from Erica Pharma, Mumbai. Crospovidone (Collidon), croscarmellose sodium (Ac-di-Sol), L-HPC and MCC (Avicel PH 102) were gift sample from Signet Chemical Corporation, Mumbai. Mannitol, camphor, sodium bicarbonate, citric acid, aspartame, and magnesium stearate were procured from Nice Chemicals (P) Ltd, Cochin. All other reagents were of analytical grade.

Mouth dissolving tablets of cinnarizine were prepared by effervescent, superdisintegrant addition and sublimation technique by direct compression method. Cinnarizine 200 mg tablets each containing 25 mg of drug were prepared. In all formulation mannitol was common diluents. The formulation of cinnarizine mouth dissolving tablet prepared by different methods is given in Table 1.

**TABLE 1:** FORMULATION AND EVALUATION OF MOUTH DISSOLVING TABLETS PREPARED BY DIFFERENT TECHNIQUE

Ingredients	Effervescent technique	Super disintegrant addition technique	Sublimation technique
Cinnarizine	25	25	25
Avicel102	50	60	-
Sodium bicarbonate	20	-	-
Citric acid	16	-	-
Crospovidone	-	15	-
Camphor	-	-	40
Mannitol up to…	200	200	200

All the quantities are in mg. All the tablets contain 1% Aspartame, 1% Mgstearate and 2% Talc

For tablets prepared by effervescent method, specified quantity of cinnarizine, mannitol, Avicel 102, aspartame, talc and magnesium stearate were weighed accurately and passed through 60 # screen. Sodium bicarbonate and citric acid were accurately weighed and preheated at a temperature of 70 °. All the materials were transferred to mortar and triturated till it mixed uniformly. The resulting powder mixture was compressed into tablets using single punch tablet machine using 9 mm flat surface punches[3].

For tablets prepared by sublimation technique, specified quantity of cinnarizine, camphor, mannitol, talc and magnesium stearate were weighed accurately and passed through 60 # screen prior to mixing. All the materials were transferred to mortar and triturated till it mixed uniformly. The resulting powder mixture was compressed into tablets using single punch tablet machine. The tablets were dried at 60 °in oven till constant weigh obtained[4].

For tablets prepared by superdisintegrant addition method, specified quantities of cinnarizine, mannitol, Avicel 102, aspartame, crospovidone, talc and magnesium stearate were weighed accurately and passed through 60 # screen. All the materials were transferred to a mortar and triturated till it was uniform. The resulting powder blend was evaluated for angle of repose, bulk density, tap density and compressibility index[5] and compressed into tablets using single punch tablet machine[6]. Tablets thus prepared using this method was given in Table 2.

**TABLE 2:** FORMULATION OF MOUTH DISSOLVING TABLETS PREPARED BY SUPERDISINTEGRANT ADDITION METHOD

Ingredient	B1	B2	B3	B4	B5	B6	B7	B8	B9
Cinnarizine	25	25	25	25	25	25	25	25	25
Crospovidone	10	15	20		-	-	-	-	-
Croscarmellose sodium	-	-	-	10	15	20	-	-	-
L-HPC	-	-	-	-	-	-	10	15	20
Avicel 102	60	60	60	60	60	60	60	60	60
Mannitol up to…	200	200	200	200	200	200	200	200	200

All the quantities are in mg. All the tablets contain 1% Aspartame, 1% Mg-stearate and 2% Talc

The prepared tablets were evaluated as per IP. Twenty tablets from each batch were weighed accurately and powdered powder equivalent to 100 mg cinnarizine was shaken with 100 ml of 0.1N HCl in 100 ml volumetric flask and from this 1 ml was pipette out and than dilute up to 100 ml. Resulting solution was filtered and assayed at 253.5 nm using double beem UV/Vis spectrometer and content of cinnarizine was calculated.

Friability (F) was determined using a Roche model friabilator and the hardness was determined using a Monsanto hardness tester (Sheetal Scientific Industries, Mumbai, India). The *in vitro* disintegration time was determined using disintegration test apparatus. The time in seconds taken for complete disintegration of the tablet with no palatable mass remaining in the apparatus was measured in seconds. Disintegration test was carried out in distilled water as medium[7]. All evaluation parameters are given in Table 3.

**TABLE 3:** EVALUATION PARAMETER OF MOUTH DISSOLVING TABLETS PREPARED BY DIFFERENT METHOD

Parameters	Effervescent	Superdisintegrant addition	Sublimation
Hardness (kg/cm^2^)	2.5	2.5	2.5
Friability (%)	0.625	0.764	0.861
Disintegration	92	34	132
time (s)			

A piece of tissue paper folded double was placed in a Petri dish (internal diameter is 6.5 cm) containing 6 ml of water. The tablet was placed on the paper, and the time for complete wetting of the tablet was measured in seconds. The method was slightly modified by maintaining water at 37°. Wetting time corresponds to the time taken for the tablet to disintegrate when kept motionless on the tongue[8].

The drug-release study was carried out using a USP XXIV type-2 apparatus (Electrolab, TDT-06T, India) at 37±0.5° and at 50 rpm using 900 ml of 0.1N HCl as a dissolution medium (n=3). A sample (5 ml) of the solution was withdrawn from the dissolution apparatus at 2, 4, 6, 8 and 10 min and withdrawn volume was replaced with fresh dissolution media. The withdrawn samples filtered through a 0.45-micrometer membrane filter, diluted suitably, and analyzed spectrophotometrically.

Various methods were tried for formulation of mouth dissolving tablets. The disintegration time of tablets prepared by various methods are shown in Table 3. It shows that super disintegration addition method exhibits the lowest disintegration time (34 s); hence it was selected for further study. The quicker disintegration time may be attributed to faster water uptake by the tablets. Friability of all batches was in the range of standard limit (less than 1%) and no more significant difference.

The angle of repose for the entire formulations blend was found to be in the range 23.49 °to 31.45 °. Formulations with crospovidone and croscarmellose sodium as a disintegrants showed angle of repose values ≤30 °where as formulation containing L-HPC showed angle of repose values > 30 °indicating only fair flow property of the powder blend. Compressibility index was found to be in the range 11.86% to 19.18%. All formulations showed good flow properties except formulation containing L-HPC 7.5% and 10% Table 4.

**TABLE 4:** EVALUATION OF PRECOMPRESSED POWDER BLEND

Batch code	Bulk density (gm/cm^3^)	Tapped density (gm/cm^3^)	Angle of repose (°)	% compressibility
B1	0.58	0.68	25.61	14.71
B2	0.56	0.67	25.07	16.42
B3	0.55	0.64	24.68	14.06
B4	0.53	0.62	24.50	14.52
B5	0.52	0.59	23.82	11.86
B6	0.50	0.57	23.49	12.28
B7	0.58	0.70	30.05	17.14
B8	0.58	0.71	30.64	18.31
B9	0.59	0.73	31.45	19.18

All the formulated (B1 to B9) tablets passed weight variation test as the % weight variation was within the IP limits of ±7.5% of the weight. The average thickness of the all formulation was found to be 2.58 mm and with in the limit of standard Pharmacopoeia. The hardness of the tablet was found to be 2.5 to 3.0 kg/cm^2^. The maximum friability of the formulation was found to be 0.8%. The minimum friability of the formulation was found to be 0.65%. The % friability was less than 1% in all the formulations ensuring that the tablets were mechanically stable. The results of drug content were within the limit specified by the IP.

*In vitro* disintegration time was found to be in the range 25.3 to 59.4 second. From all formulations, B9 (10% L-HPC) has minimum time of disintegration. Formulations containing croscarmellose sodium has taken more time for disintegration because of its gelling properties. Wetting time was found to be in the range 29.1 to 89 s. From all formulations, B9 (10% L-HPC) has minimum wetting time (Table 5). All the 9 formulations were subjected to *in vitro* dissolution studies by using 0.1N HCl. Dissolution data shows that formulation B9 shows improved dissolution as compared to other formulations and more than 90% drug release was found at the end of 4 min (fig. 1).

**TABLE 5:** EVALUATION OF MOUTH DISSOLVING TABLET OF CINNARIZINE

Batch code	*In vitro* disintegration Time (s)*	Wetting time (s)*	Assay (%)
B1	48.3±.53	69.8±1.04	98.14
B2	34.0±1.00	35.0±0.95	99.02
B3	28.6±.22	32.4±1.15	100.51
B4	59.4±2.42	89.0±0.85	98.91
B5	32.6±1.25	66.0±1.35	100.04
B6	36.6±2.12	70.4±1.48	99.86
B7	59.7±2.46	67.8±0.35	98.92
B8	33.5±0.50	41.7±1.45	101.05
B9	25.3±0.58	29.1±1.05	100.34

* (n= 3)

**Fig. 1:**
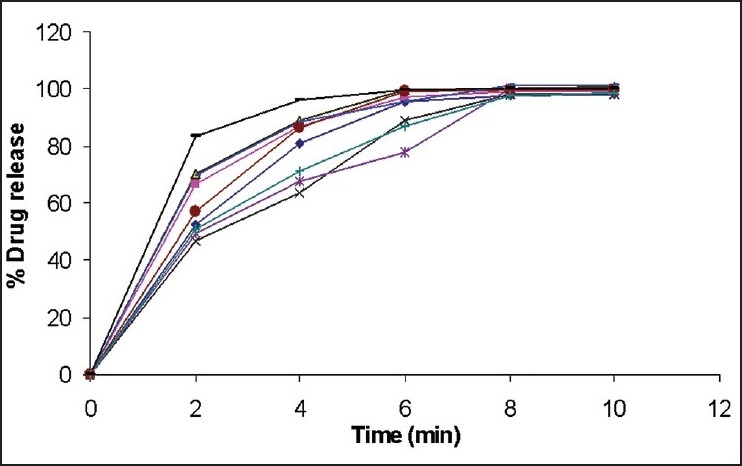
Drug release profile of mouth dissolving tablets of Cinnarizine Batch B1 (—♦—), Batch B2 (—■—), Batch B3 (—▲—), Batch B4 (—× —), Batch B5 (—*—), Batch B6 (— ● —), Batch B7 (—+—), Batch B8 (—_—), Batch B9 (— __ —)

In the present work, mouth dissolving tablets were prepared by effervescent, superdisintegrant addition and sublimation technique by direct compression method, from all these techniques, superdisintegrant addition technique was selected based on least disintegration time.

The mouth dissolving tablets of cinnarizine were prepared by superdisintegrants addition method using crospovidone, croscarmellose sodium and L-HPC in different concentration like 5%, 7.5% and 10%. There are total nine formulations were prepared and evaluated for various parameters. Formulation B9 containing L-HPC in concentration of 10% showed minimum disintegration time, wetting time as compare to other formulations. Results of dissolution studies showed that total drug was released in 6 min. The results shown that disintegration time was increased in the manners of L-HPC< crospovidone< croscarmellose sodium.
